# Studies on the Infection, Colonization, and Movement of *Pseudomonas syringae* pv. *actinidiae* in Kiwifruit Tissues Using a GFPuv-Labeled Strain

**DOI:** 10.1371/journal.pone.0151169

**Published:** 2016-03-21

**Authors:** Xiaoning Gao, Qiling Huang, Zhibo Zhao, Qingmei Han, Xiwang Ke, Huqiang Qin, Lili Huang

**Affiliations:** College of Plant Protection and State Key Laboratory of Crop Stress Biology for Arid Areas, Northwest A&F University, Yangling, Shaanxi, P.R. China; Virginia Tech, UNITED STATES

## Abstract

Kiwifruit bacterial canker, an economically important disease caused by *Pseudomonas syringae* pv. *actinidiae* (*Psa*), has caused severe losses in all major areas of kiwifruit cultivation. Using a GFPuv-labeled strain of *Psa*, we monitored the invasion, colonization, and movement of the pathogen in kiwifruit twigs, leaves and veins. The pathogen can invade twigs through both wounds and natural openings; the highest number of *Psa* is obtained in cut tissues. We determined that, following spray inoculation, *Psa*-GFPuv could infect leaves and cause lesions in the presence and absence of wounds. Light and transmission electron microscopic observations showed that bacterial cells colonize both phloem and xylem vessels. Bacterial infection resulted in marked alterations of host tissues including the disintegration of organelles and degeneration of protoplasts and cell walls. Furthermore, low temperature was conducive to colonization and movement of *Psa*-GFPuv in kiwifruit tissues. Indeed, the pathogen migrated faster at 4°C than at 16°C or 25°C in twigs. However, the optimum temperature for colonization and movement of *Psa* in leaf veins was 16°C. Our results, revealing a better understanding of the *Psa* infection process, might contribute to develop more efficacious disease management strategies.

## Introduction

Kiwifruit (*Actinidia deliciosa*, the green-fleshed kiwifruit and *A*. *chinensis*, the yellow-fleshed kiwifruit), native to China, is an economically important fruit crop. In recent years a bacterial canker of kiwifruit, caused by *Pseudomonas syringae* pv. *actinidiae* (*Psa*), has caused severe losses in all major areas of kiwifruit cultivation. The pathogen was first reported and described in Japan in 1989 [1]; then, it was detected in Korea [2], Italy [3] and China [4]. In recent years, *Psa* re-emerged in all countries where *A*. *deliciosa* and *A*. *chinensis* are cultivated, such as in Japan, Korea, Italy, France, Portugal, Chile, Spain, Turkey and New Zealand [5]. In Italy, the yield losses caused by *Psa* infection were estimated to cost up to 2 million Euros in the spring of 2008 [6]. In New Zealand, bacterial canker caused by the virulent strain of *Psa*, has seen predicted to cause yield losses between $310 and $410 million over the next five years, which will probably increase to $740-$885 million over the next 15 years [7]. In China, *Psa* was first described in Sichuan province [4], then the pathogen spread soon to other regions of China where kiwifruits were grown, such as in the provinces of Shaanxi, Sichuan, Hunan and Hubei. In these areas kiwifruit plantations have also been severely affected by the disease. In Shaanxi, the largest kiwifruit producer in China, the *Psa* infected area expanded from 0.13 to 311.5 ha between 1992 and 2002 [8]. In 2012, up to 80% of the orchards were affected, and the portion of diseased plants at each site ranged from 30% to 50% [9].

*Psa* can infect the above ground organs of kiwifruit trees. In green-fleshed and yellow-fleshed kiwifruit cultivars, the symptoms include canker formation on the trunk and vines with whitish to red exudates, brown discoloration of twigs, leader and cane dieback, reddening of lenticels, brown-black leaf spots with yellow halos, and blossom necrosis [6]. A survey of previous *Psa* studies revealed that they can be divided into two categories. Firstly, in the 1990s, studies were carried out mainly in Japan and China and focused on isolation and identification of the pathogen in kiwifruit orchards [1,2,4,10], as well as on disease prevalence and possible effects of environmental factors on the disease development [1,11,12,13,14,15]. Secondly, during this period the efficacy of different chemicals, including copper compounds and streptomycin, were investigated to control *Psa* [16,17]. Since the “global outbreak of Psa in 2008”, great efforts have been made to better understand the nature of the disease. For example, PCR-based methods have been used to detect the survival and transmission of *Psa* [18,19,20,21,22]. Additionally, the global populations of *Psa* have been redefined based on pathogenic, molecular, and phenotypic characteristics [23,24,25,26,27]. Whole genome sequencing analyses have been performed to understand specific traits of *Psa* determining processes and factors of its prevalence and virulence [28,29,30]. These studies have made important contributions to our understanding of the re-emergence of *Psa*, and have suggested strategies for protection of *Psa*-threatened crops [31].

However, our knowledge on mechanisms determining infection, colonization and movement processes of *Psa* are still insufficient. In a previous study, we engineered a *Psa* strain expressing the fluorescent protein, GFPuv [32]; this approach greatly facilitated monitoring of *Psa* during colonization and movement in plant tissues of kiwifruit. In the present study, we combined the fluorescence monitoring method with electron microscopy, in order to elucidate in more detail infection colonization and spread of *Psa* at different temperatures in various plant tissues of kiwifruit.

## Materials and Methods

### Bacterial strain, inoculum preparation, and plant material

Strain Psamx7 was isolated in Mei county of Shaanxi province from infected leaves of *A*. *chinensis* ‘Hongyang’, which is widely cultivated in China and susceptible to *Psa*. No specific permissions were required for collecting sample, because ‘Hongyang’ is widely cultivated in Mei county of Shaanxi province and *Psa* infected leaves were observed in every kiwifruit orchard. Furthermore, we confirm that the field studies did not involve endangered or protected species. The strain was identified as *Psa* biovar 3 according to the concatenated 16S-gyrB-dnaA sequence [22]. This strain was successfully transformed with GFPuv by electroporation [32]. Compared to the wild *Psa* strain, the modified strain Psamx7-GFPuv1 (named as Psa-GFPuv) showed no significant differences regarding morphology, growth pattern and pathogenicity; since expression level of GFPuv in the modified strain is also stable and strong during cell multiplication [32], the GFPuv-labeled strain is suitable not only for studying colonization and movement of *Psa* but also to trace formation and spread of lesions in kiwifruit leaves and veins.

The modified strain, was routinely grown for 48 h at 28°C on beef peptone agar (BPA) medium (Joel High-Tech Co., Ltd., Dalian, China) (containing tryptone 1%, beef extract 0.3%, yeast extract 0.1%, sucrose 1%, agar 1.5%, pH 7.0) and supplemented with kanamycin (KAN, 50 μg·mL^−1^). Then, single colonies were picked and cultured in BP liquid medium supplemented with KAN for 16–20 h at 28°C on a rotary shaker (180 RPM). For inoculation, the culture was centrifuged at 8000× *g* for 20 min and the bacterial cells were re-suspended in phosphate buffer solution (PBS, pH 7.0). Bacterial suspensions, diluted to 2×10^7^ CFU·mL^-1^, were used to inoculate kiwifruit twigs and leaves.

Two-year old kiwifruit trees of *A*. *deliciosa* ‘Xuxiang’ were grown and maintained in the greenhouse. Temperature was maintained at 25°C for 14 h by light and at 15°C for 10 h in the dark; relative humidity (RH) was maintained between 60 and 70%.

### Inoculation in kiwifruit tissues with *Psa*

#### Twig inoculation

To ascertain whether the method of wounding affects invasion and colonization of Psa-GFPuv, we created different injuries prior to inoculation, including inoculation by smearing unwounded lenticels, inoculation by dripping twigs after causing slight injury (to simulate natural rubbing), inoculation by smearing leaf scars, and inoculation by dripping after cutting. Approximately 1 cm above the inoculation sites, twigs were covered with moist sterile cotton and plastic wraps for 3 day. Each plant was inoculated once at a single inoculation site, and twigs treated correspondingly with PBS were used as controls. Each experiment consisted of three replicates of the different inoculation types and the experiment was repeated three times. The treated plants were kept in growth chambers with cycles of 14 h light and 10 h darkness. During the experiments, plants were maintained at 95% RH.

#### Inoculation of leaves and veins

Before inoculation, fully expanded kiwifruit leaves were rinsed three times by spraying with sterile water. Then, the bacterial suspension (2×10^7^ CFU mL^-1^) was sprayed onto the upper and lower surface of non-wounded kiwifruit leaves using a hand-held sprayer. Leaf veins were inoculated through wounds using a sterile needle and then a drop of the bacterial suspension applied on the wound sites. In each experiment the different inoculation types were repeated five times and the experiment was repeated three times.

### Quantitative analysis of *Psa* in kiwifruit twigs

In order to determine colonization of Psa-GFPuv in twigs, samples were collected at 3, 6, 24, and 72 HAI (hours after inoculation). For isolation, samples were surface sterilized by dipping in 70% ethanol for 5 min followed by immersion in 1% (wt/vol) Clorox for further 5 min and then washed thrice in sterile water and blotted with sterile filter paper. Samples were thoroughly ground in 5 mL of 0.1 M PBS. Serial dilutions (2 × 10^−4^, 10^−5^, 10^−6^, and 10^−7^) of bacterial suspensions (100 μL) were placed directly on BPA plates supplemented with KAN. Three plates per dilution were used. After incubation at 25°C for 2 d, the plates were illuminated with UV light and GFP-expressing colonies were counted. Bacterial cell numbers were calculated on the basis of the fresh weights of twig tissues (CFU·g^-1^ tissue).

### Microscopic observation

For microscopy studies, leaves, veins and twigs were similarly inoculated as described above. The kiwifruit plants with inoculated twigs were cultivated in a chamber at 4°C and those plants with inoculated leaves were kept of 16°C. To observe Psa-GFPuv colonization in kiwifruit tissues by light and transmission electron microscopy (TEM), from each inoculation type 5 samples of twigs were collected 7, 15 and 25 DAI (days after inoculation), and leaf samples were taken 5 and 10 DAI. Samples were cut into small pieces (5 × 3 mm^2^) and processed for light microscopy and TEM as described by Kang and Buchenauer [33] and Ke *et al*. [34]. For TEM analysis, ultrathin sections of samples cut with a diamond knife were collected on copper grids. The grids were examined with a TEM 1230 microscope (JEOL) after contrasting with uranyl acetate and lead citrate.

### Effects of temperature on the spread of *Psa*

#### Twig inoculation and treatment

The inoculation method followed the procedure described by Zhao *et al*. [9] with minor modifications. The twigs were subjected to one oblique cut with a sterile scalpel, and the wound was inoculated with a 40-μL droplet of bacterial suspension. To determine the effect of temperature on colonization and spread of Psa-GFPuv, the treated kiwifruit plants were placed at 4°C, 16°C, and 25°C in growth chambers and RH was maintained at 95%. The samples were collected every 3 for 24 HAI, then each day for the next 10 d, and thereafter every second day until 30 d. Starting at the inoculation site, tissues were cut in 2 cm sections for a total distance of 15 cm. The methods of isolation and quantification were the same as described above.

#### Leaf vein inoculation and treatment

Leaf veins were inoculated as described above. After inoculation plants were cultured in chambers at 16°C, 20°C, and 24°C at RH>95%. To visualize bacterial infection and migration in leaf veins, the treated leaves were directly examined and photographed under a fluorescence stereomicroscope (Leica MZ12) in a dark room each day after inoculation.

## Results

### Invasion and colonization of *Psa-*GFPuv in twigs kiwifruit tissues

#### Twigs

**Fig 1:**
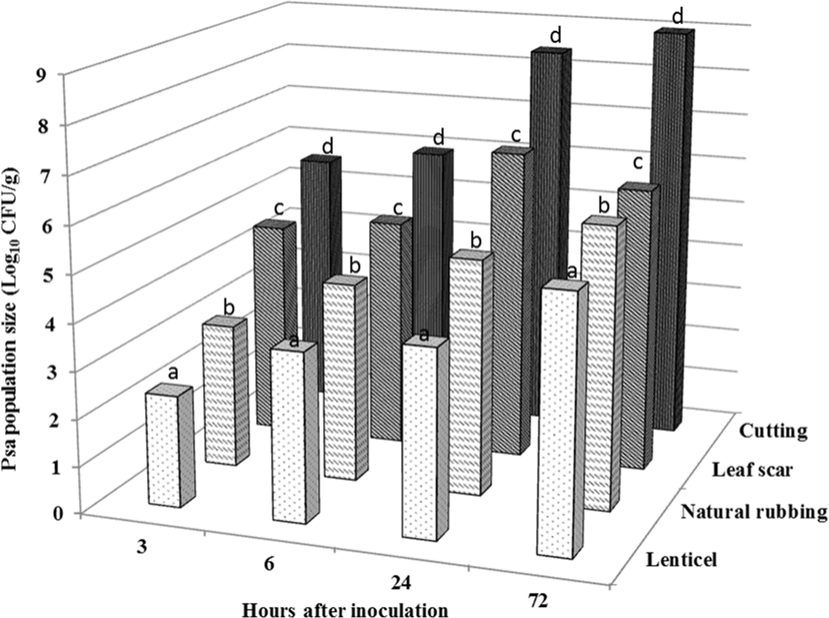
Population size of the GFPuv-labeled *Psa* in kiwifruit twigs after inoculation of different wound types. The GFPuv-labeled *Psa* were isolated and cultured in BPA plates supplemented with KAN (50 μg·mL^−1^) and colonies were counted under the fluorescence microscope. Values in columns followed by different letters were significantly different according to Fisher’s protected LSD test among lenticel, natural rubbing, leaf scar and cutting (P = 0.05).

Although the pathogen was isolated from all experimental samples, the relative number of fluorescent colonies varied according to the inoculation site and/or wound type (Fig 1). In lenticels, 3 HAI 2.4±0.5×10^2^ CFU·g^-1^ were determined; the number of bacterial colonies was significantly lower than in samples of leaf scar and in cut tissues (4.2±0.3×10^4^ CFU·g^-1^ and 3.8±0.3×10^5^ CFU·g^-1^, respectively). Between 3 and 72 HAI, the number of fluorescent colonies in the plant tissues exponentially increased. The highest number of colonies was obtained in cut tissues 72 HAI (9.9±0.2×10^8^ CFU·g^-1^). At the same incubation period (72 HAI), the relative number of colonies in samples of lenticels, naturally rubbed tissues, and leaf scars were 2.2±0.4×10^5^, 9.4±0.2×10^5^ and 1.2±0.5×10^7^ CFU·g^-1^, respectively. The results suggest that GFUuv-labeled *Psa* strain can enter via different wound types and natural openings.

#### Population dynamics of *Psa-*GFPuv in twigs

**Fig 2:**
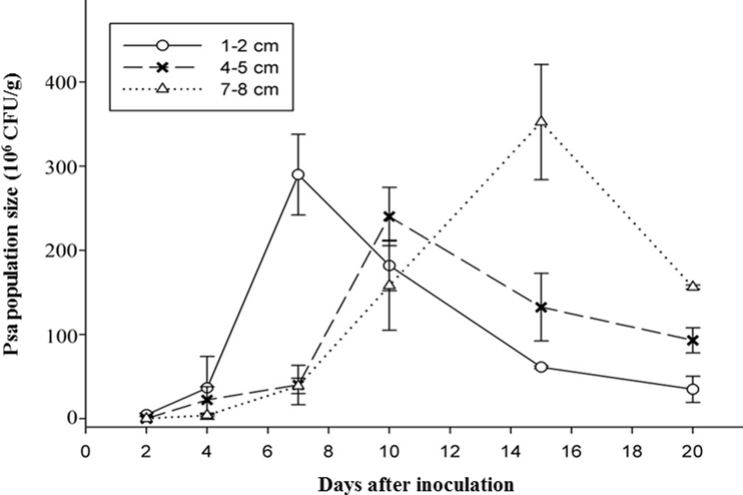
Quantification of the GFPuv-labeled *Psa* in kiwifruit twigs using the plating method (in BPA plates supplemented with 50 μg·mL^−1^KAN) following cutting inoculation.

Population of the labeled *Psa* strain was quantified in different sections (1–8 cm) from the cut inoculation site of twigs during an incubation time of 2 to 20 days. In each twig section, bacterial cells were detected (Fig 2). After inoculation, bacterial colonies increased more rapidly in the section close to the inoculation site, whereas, increase in colony numbers was delayed in the middle and more remove twig section. At 1–2 cm from the inoculation site, the number of Psa-GFPuv reached 3.1×10^6^ CFU·g^-1^ at 2 DAI; then, a constant increase until a maximum of 2.9×10^8^ CFU·g^-1^ was reached 7 DAI. Similar population dynamics were observed in section 4–5 cm and 7–8 cm from the inoculation site; the highest population numbers in the middle and more distant sections were reached 10 and 15 DAI. After maximum of bacterial population was reached, colony numbers in each section decreased.

#### Invasion and colonization of *Psa* in kiwifruit leaves

**Fig 3:**
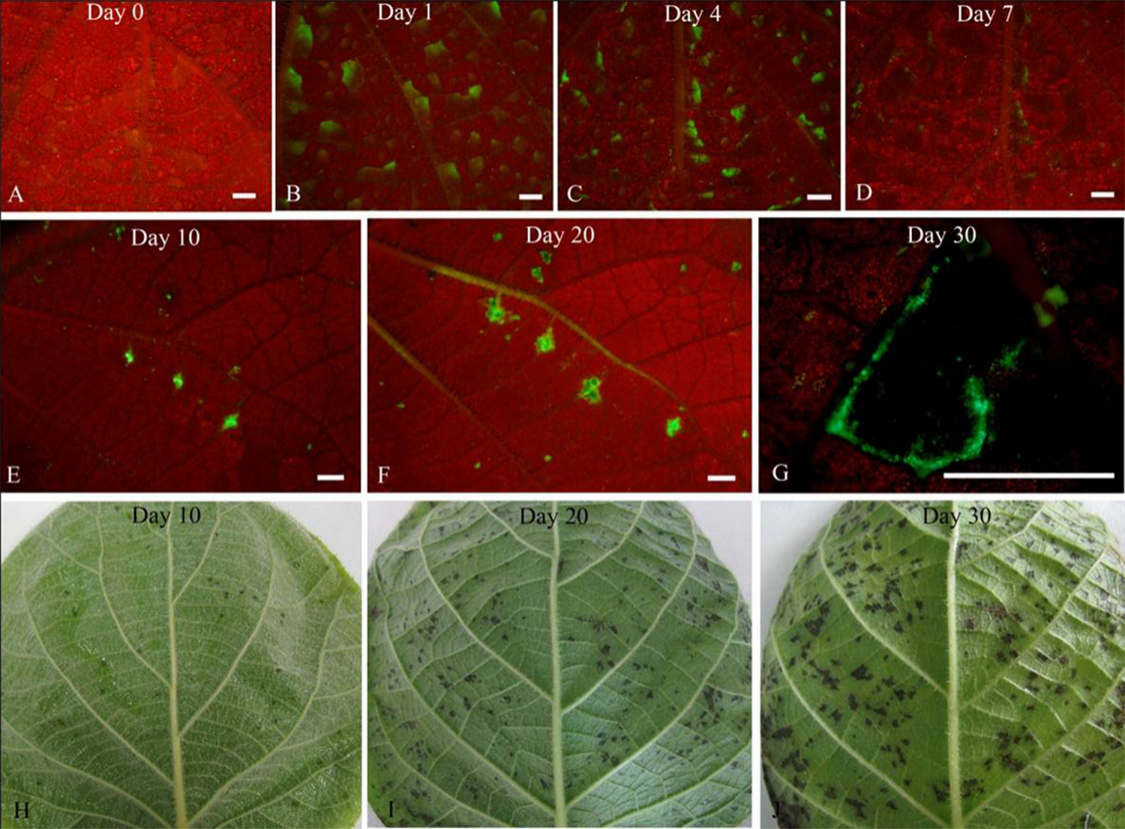
Formation and spread of the leaf lesions by spraying the GFPuv-labeled *Psa*. The inoculated leaves were observed under a fluorescence stereomicroscope (Leica MZ12) at different days after inoculation. Bar = 2 mm

The colonization and spread of Psa-GFPuv was observed in kiwifruit leaves by using fluorescence stereomicroscopy (Fig 3). After spraying bacterial cells onto non-wounded leaf surfaces, green fluorescence (Psa-GFPuv strain) was clearly observed in the inoculated leaves; the fluorescence intensity gradually increased and reached a maximum at 4 DAI. At 7 DAI, green fluorescence became steadily weaker. Concomitant with the appearance of brown lesions at 10 DAI, there was an increase in the size of green fluorescent zones surrounding the lesions. Following extension of lesions, the size of the green fluorescence area increased and was even more intense than in veins at 20 DAI. However, at 30 DAI, the intensity of green fluorescence at the margin of lesions clearly decreased.

### Microscopical observations

#### Infection of twigs by *Psa*

**Fig 4:**
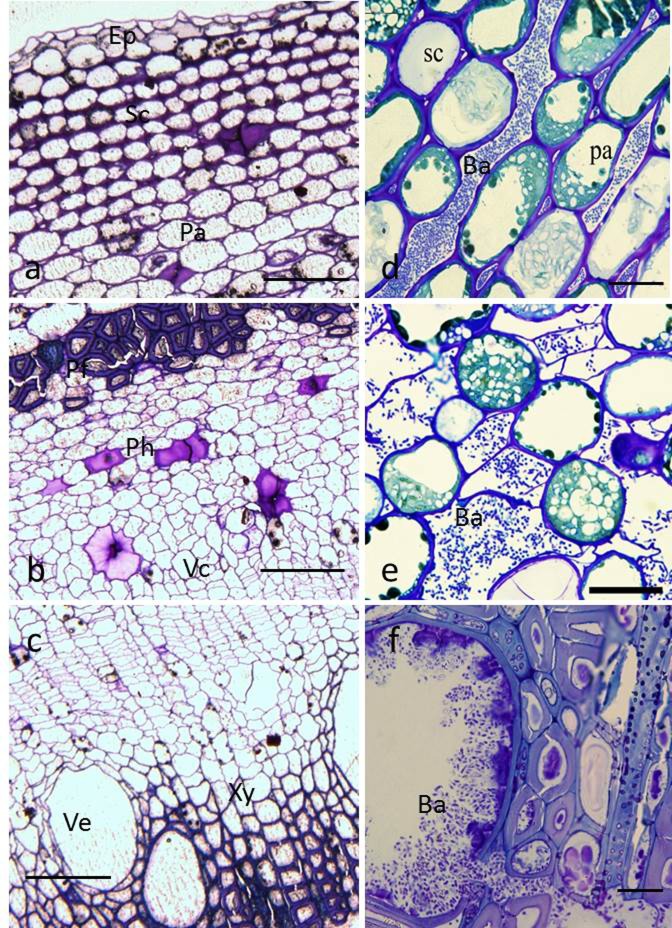
Light micrographs of healthy and infected kiwifruit bark. (a–c), Transverse sections of healthy bark, showing typical anatomical tissues consisting of a typical epidermis (Ep), sclerenchyma (Sc), parenchyma (Pa), phloem fiber (Pf), phloem (Ph), vascular cambium (Vc), xylem (Xy), and vessel (Ve). Bar = 100 μm. (d–f), transverse sections of inoculated bark. (d), bacterial cells (Ba) in intercellular spaces of parenchyma; (e), bacterial cells in phloem cells and intercellular spaces; (f), bacterial cells tightly packed within xylem vessels. Bar = 20 μm.

Light microscopic observations of thin sections of the non-infected kiwifruit twigs revealed the typical anatomy of kiwifruit bark tissue, consisting of epidermis, sclerenchyma, parenchyma (Fig 4A), phloem fiber, phloem, vascular cambium (Fig 4B), and xylem and xylem vessel (Fig 4C). Seven DAI of cut wounds, the pathogen had extensively colonized the intercellular parenchymal spaces (Fig 4D). Accompanied with lesion development, high bacterial cell numbers were observed in phloem cells and intercellular spaces (Fig 4E), and also in xylem vessels at 15 DAI (Fig 4F).

**Fig 5:**
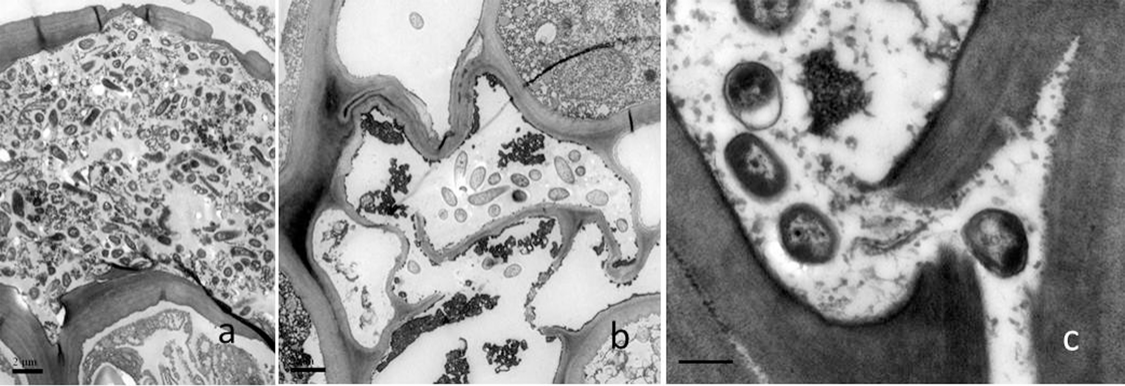
Transmission electron micrographs of cross sections of kiwifruit twigs infected with the GFPuv-labeled *Psa* 5 DAI. (a), Bacterial cells in intercellular spaces of the parenchyma. Bar = 2 μm; (b), bacterial colonization in parenchyma cells where the cell walls are ruptured and organelles have disintegrated. Bar = 2 μm; (c), bacterial colonization in intercellular spaces where the middle lamella tended to be ruptured. Bar = 0.5 μm.

TEM observation of infected kiwifruit bark showed that host cells were aggressively colonized by GFPuv-labeled *Psa*. Five DAI, bacterial cells have been predominantly located in the intercellular space between cortical and parenchyma cells (Fig 5A). During Psa-GFPuv colonization, host cells tended to rupture and exhibited signs of organelle disintegration (Fig 5B and 5C).

#### Detection of *Psa*-GFPuv in leaves and veins

**Fig 6:**
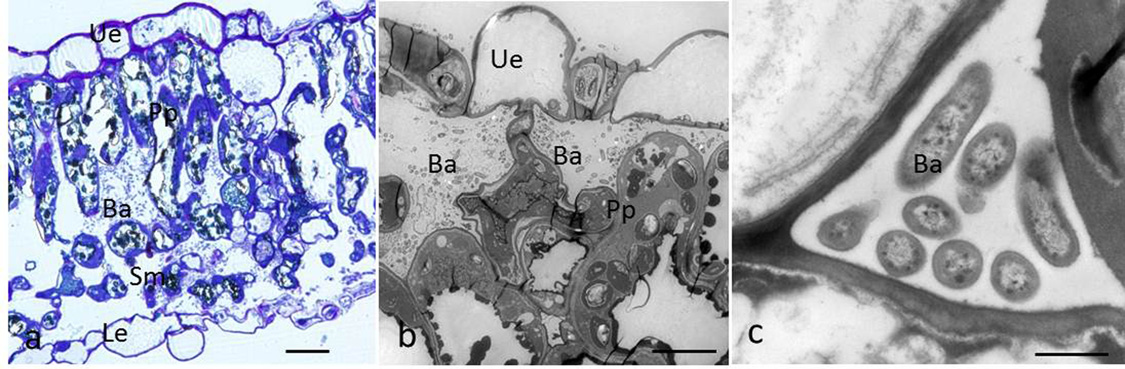
Light and transmission electron micrographs of a cross-section of kiwifruit leaf infected by the GFPuv-labeled *Psa*. (a), Intercellular bacteria in the mesophyll cells at 5 dai. Bar = 50 μm; (b), bacterial colonization in the palisade parenchyma where the cells were abnormal. Bar = 10 μm. (c), bacteria that have colonized the intercellular space. Bar = 1 μm. Abbreviations: upper epidermis (Ue); palisade parenchyma (Pp); spongy mesophyll (Sm); lower epidermis (Le); bacteria (ba).

Sections of leaves inoculated by spraying demonstrated that bacterial cells massively accumulated in mesophyll cells of the foliage at 5 DAI (Fig 6A). High-magnification images revealed that bacterial cells were embedded within epidermis cells, palisade parenchyma, and the intercellular space (Fig 6B and 6C). During leaf infection, morphology of mesophyll cells changed due to the presence of very high numbers of bacteria that invaded the intercellular space (Fig 6B and 6C).

**Fig 7:**
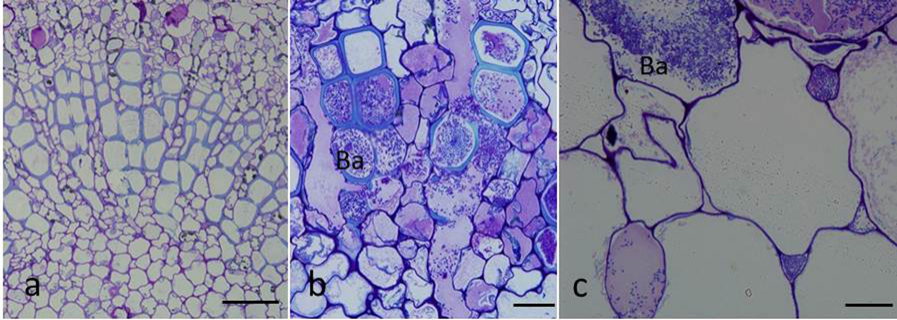
Light micrographs of leaf veins colonized by GFPuv-labeled *Psa* cells. (a), Cross-section of the healthy vein. Bar = 50 μm; (b), bacterial colonization of vessels in the vascular bundle. Bar = 20 μm; c, bacterial colonization of the cortex cells and intercellular spaces. Bar = 20 μm.

The vessels of the vascular bundle, cortex cells, and intercellular spaces were infected by *Psa* (Fig 7B and 7C). In contrast to the findings for healthy veins (Fig 7A), pathogen-induced morphological alterations, such as ruptured cell walls, were seen in infected host cells (Fig 7B).

### Effects of temperature on the spread of *Psa*-GFPuv in kiwifruit tissues

#### Effects of temperature on spread of bacterial cells in twigs

**Fig 8:**
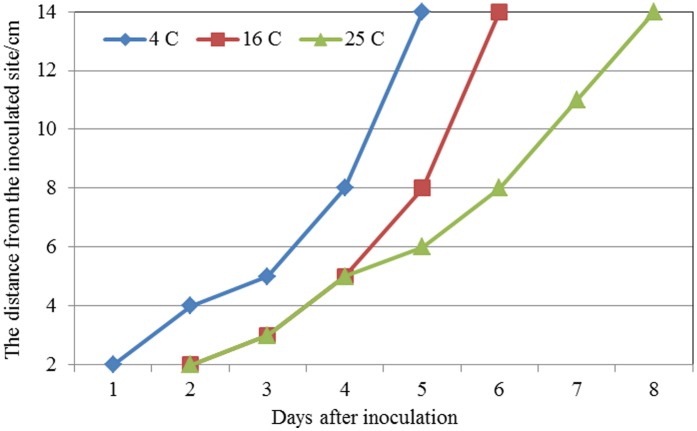
The migration of the GFPuv-labeled *Psa* in kiwifruit twigs in different temperature treatments by cutting inoculation. Samples were taken 2 cm from the inoculation site. The GFPuv-labeled *Psa* were isolated and cultured in BPA plates supplemented with KAN (50 μg·mL^−1^) and colonies were counted under the fluorescence microscope.

The data showed that temperature had a significant effect on the movement of the pathogen in kiwifruit twigs (Fig 8). Specifically, the rate of spreading is faster at 4°C compared to inoculation at 16°C or 25°C. Fluorescent colonies were isolated at a distance of 2 cm from the inoculation site 1 DAI when the inoculated kiwifruit twigs were incubated at 4°C; by contrast, it took 2 d for colonies to reach this distance when tissues were placed at 16°C and 25°C. A similar effect of temperature was noticed at the more distal sites (14 cm from the inoculation site). For example, Psa-GFPuv reached this distance in 5 DAI at 4°C, but took 6 d or 8 d when tissues were kept at 16°C or 25°C, respectively.

#### Effects of temperature on the spread of Psa-GFPuv in leaf veins

**Fig 9:**
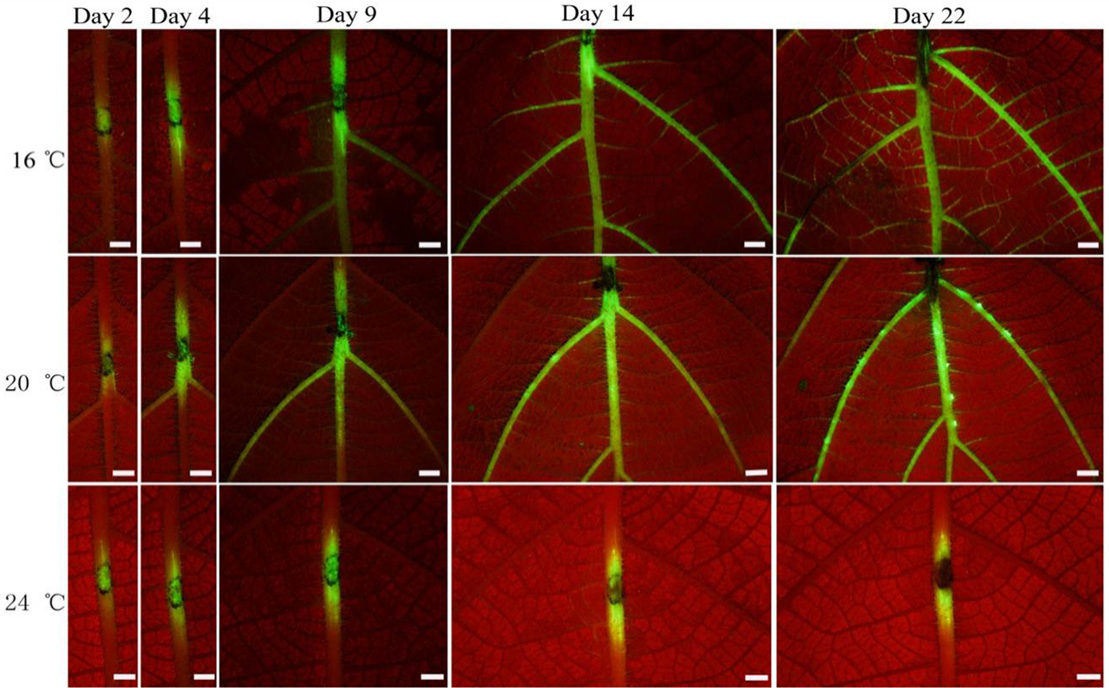
The GFPuv-labeled *Psa* colonization and migration in kiwifruit leaf veins. The inoculated leaves were observed under a fluorescence stereomicroscope (Leica MZ12) at different days after inoculation. Bar = 2 mm.

Bacterial cells could invade and colonize veins after wounding with a sterile needle; however, the incubation temperature influenced the degree of bacterial cells spreading. While green fluorescence was observed in the entire main vein and lateral veins 14 DAI at 16°C, it was present only in the main vein at 20°C (Fig 9). Increasing the temperature to 24°C, colonization of veins by the pathogen was clearly restricted to a narrow distant of the inoculation sites.

## Discussion

Weather conditions that favor bacterial infection are usually sporadic, being confined to particular seasons and are associated with rainy, wet and humid conditions. Once plants are infected, disease development is largely determined by temperature [35]. *Psa* is reported to cause disease at relatively low temperatures in kiwifruit [13,14]. Cankers in trunks and leaders are the most serious symptoms, which develop most rapidly in late winter and spring, because they girdle whole limbs and kill entire vines [15,36]. Serizawa et al. (1993) tested the effect of temperature from 10°C to 25°C on the disease development on new canes. These results suggested that the optimum temperature for growth of *Psa* on new canes was in the range of 10 to 20°C [14]. *Psa* populations in infected host tissues decreased when temperatures increased and, at temperatures above 25°C, *Psa* inoculations did not lead to exudate formation or infection in canes [14]. In Italy, *Psa* in cankers of kiwifruit trees usually die out in spring to summer when temperatures rise above 20°C [35]. This is not the case in New Zealand, which has a mild maritime, practically frost-free, climate; disease in canes is practically unchecked, the disease spreading in canes throughout the summer [35]. In our study, we found that the GFPuv-labeled *Psa* strain could invade and grow in the temperature range between 4 and 25°C in twigs. Epidemics occur under precisely prescribed conditions, usually appearing regionally or sub-regionally. According to our results, several important findings about the behavior of *Psa* could be found in terms of Chinese seasonal conditions compared with those in other countries. Only when the pathogen is distributed more widely in areas with a wider climatic range, it will be to establish possible in these areas for future planting and those to avoid.

However, to emphasize an important point, the result of the growth curve of *Psa* showed that the pathogen could grow *in vitro* in a temperature range from 4 to 35°C, the optimum temperature was at 25°C [32]. Obviously there is a great temperature difference between growth of *Psa in vitro* and *in vivo*. The main reason is that growth of the bacterium in the host tissue was abruptly inhibited at high temperature by the formation of wound-healing tissue surrounding the infected area. Serizawa et al. [15] reported that formation of wound healing tissue was highest in mid-summer (with a mean temperature of 25°C). Formation of wound-healing tissue gradually ceased when the temperature dropped gradually from 22 to 20°C; and no healing of the affected tissue was observed when the temperature dropped to 15°C or below. In New Zealand, researchers [37] found a similar relationship between spread of *Psa* and temperature in kiwifruit tissues. More rapid and complete healing occurred on pruning cuts made in late spring and summer than on early spring cuts. This study also showed that bacterial lesions were confined where callus formation rapidly occurred.

Several researchers reported that dark angular necrotic spots in leaves were often accompanied by yellow chlorotic halos around the outer edge of the spots, especially in early spring and summer [35,38,39]. In Japan, Serizawa and Ichikawa [14] found that bacterial populations in leaf lesions were highest in late spring and autumn; when the mean temperature over a period of 10 days prior to isolation ranged between 20°C and 24°C, but, *Psa* populations dropped rapidly during summer. Once the temperature increased over 25°C, *Psa* could either not be detected in some lesions or in other lesions bacterial population was very low. To date, there is very little information about the effect of temperature on the spread of *Psa* in kiwifruit leaf veins. Using fluorescence stereomicroscopy, we visualized spread of bacteria in veins at 16 to 25°C. At the lower temperature (16°C), *Psa* rapidly spread from the inoculated site to the main and lateral veins. At 25°C, fluorescence was restricted close to the inoculated site.

*Psa* appeared to be capable colonizing the host all year round and to migrate systemically from young leaves to twigs. Unprotected lenticels, fruit stalks and leaf scars, as well as pruning wounds and tissues surrounding the elastic laces were easily colonized by the pathogen [40]. Consistent with previous reports on the routes by which pathogenic bacteria enter plants [37,41], we found that *Psa* could enter the twigs though natural openings and wounds. Strikingly, however, we found that the type of wound has a remarkable influence on the amount of bacterial colonization. Of all wound types studied, cutting was the most favorable for *Psa* to colonize the host tissues. Within this context, some agronomical techniques (i.e., pruning, tying of young twigs) causing wounds to the tree greatly contribute to increase the possibility of colonizing of the plant [37].

Using microscopic techniques, we were able to localize the pathogen and to observe multiplication and accumulation of *Psa* cells in the twig, leaf, and vein tissues; this approach also allowed us to monitor the host response. Through microscopic examination, *Psa* cells were present in all regions of the cane down to the cambium layer in two *A*. *chinensis* cultivars; but no bacterial cells were detected in the xylem or woody parts of the cane, however, in heavily infected canes in some samples bacterial cells were found [37]. We also showed that the GFPuv-labeled *Psa* strain colonized twigs intensively after wound inoculation; especially, high bacterial cell numbers were observed in xylem vessels. Colonization of xylem and phloem vessels may explain that the pathogen can spread systemically to young twigs within few minutes after penetration. Furthermore, Psa-GFPuv could effectively colonize internal portions of kiwifruit twigs and, subsequently, migrate to the leader and main trunk during the following season [42]. In addition, *Psa* bacterial cells also colonized the intra- and intercellular spaces, and this activity was associated with rupture of the cell walls of the plant tissue [43–44]. The pathogenesis factors of *Psa* causing cell wall ruptures will be investigated in future studies.

In conclusion, we suggest that prevention of wounds or protecting them as well as a temperature-controlled environment should form part of new strategies aimed at better management of kiwifruit orchards. Our fluorescent tracking system can be further exploited to test other environmental or man-made factors for the effects on the pathogenicity of bacterial plant pathogens or resistance of new cultivars.

Author Contributions: conceived and designed the experiments: XG LH

Author Contributions: performed the experiments: XG QLH ZZ HQ

Author Contributions: analyzed the data: XG QMH

Author Contributions: contributed reagents/materials/analysis tools: QLH XK

Author Contributions: wrote the manuscript: XG QLH LH
